# Association between bone turnover markers, BMD and height loss of cemented vertebrae after percutaneous vertebroplasty in patients with osteoporotic vertebral compression fractures

**DOI:** 10.1186/s13018-022-03087-4

**Published:** 2022-04-04

**Authors:** Shangjin Lin, Xiaoxi Cai, Qun Cheng, Cong Chen, Xuhai Cao, Fengjian Yang, Yongqian Fan

**Affiliations:** 1 Department of Orthopeadic, Huadong Hospital Affiliated to Fudan University, Shanghai, 200040 China; 2 Department of Osteoporosis and Bone Disease, Huadong Hospital Affiliated to Fudan University, Shanghai, 200040 China

**Keywords:** BMD, Percutaneous vertebroplasty, 25-OH-D3, Osteoporotic vertebral compression fracture, Cemented vertebra recompression

## Abstract

**Introduction:**

Percutaneous vertebroplasty (PVP) was recently performed for treating patients with osteoporotic vertebral compression fractures (OVCF). However, recompression of cemented vertebra with significant vertebral height loss occurred in the patients after PVP was observed during the follow-up period. The purpose is to explore the risk factors among several potential predictors for the height loss of treated vertebral bodies after PVP in patients with OVCF.

**Methods:**

A study of 93 patients who had undergone PVP between May 1, 2016, and March 1, 2019, at the Spine Center of Huadong Hospital Affiliated to Fudan University was conducted. The fractured vertebral height loss ratio ≥ 15% at final follow-up were defined as cemented vertebra recompression. The following variables were measured and collected: age, gender, body mass index (BMI), bone mineral density (BMD), volume of bone cement injected, bone cement leakage, fractured vertebra segment, contact between bone cement and endplates, serum of calcium and phosphorus, and six kinds of bone turnover markers.

**Results:**

Mann–Whitney U test and Univariate Logistic regression analysis showed that the cemented vertebra recompression was correlated with BMD, contact between bone cement and endplates, parathyroid hormone (PTH), and 25-hydroxy vitamin D3 (25-OH-D3). Following multivariate modeling, multiple factors logistic regression elucidated that high BMD (*P* < 0.001, OR = 0.089) and high level of serum 25-OH-D3 (*P* = 0.012, OR = 0.877) were negatively correlated with the cemented vertebra recompression after PVP.

**Conclusion:**

Decreased BMD and lower level of serum 25-OH-D3 might be two critical and significant risk factors for the height loss of cemented vertebrae after PVP.

## Introduction

Under the background of population aging in China, osteoporotic vertebral compression fractures (OVCF) have a significant impact on patients’ quality of life, which has been frequently developed in patients with osteoporosis or osteopenia [1, 2]. The conservative treatments for OVCF include non-weight-bearing bed rest, orthosis use, and drug treatment. The use of spinal orthoses plays a certain role in maintaining spinal stability and limiting progression of the deformity [3]. However, despite proper conservative treatment, OVCFs also result in some complications among the elderly population, such as deep vein thrombosis, progressive kyphosis, impaired gait, decreased pulmonary function, and urinary system infection [4].

As these problems in lives decline in the quality of life, a more effective treatment should be created. Percutaneous vertebroplasty (PVP), used to treat vertebral angioma, was initially introduced in the radiology literature in 1987 [5]. Then PVP is widely used to treat back pain associated with OVCF, as it makes significant pain relief, and patients get the early mobilization [6]. It is generally accepted as a minimally invasive technique, which can structurally stabilize a fractured vertebral body through the injection of self-curing cement substance and make practical importance for reducing the back pain due to the OVCF. During follow-up of our patients, however, we observed the height loss of augmented vertebrae after PVP, which might result in the aggravation of local kyphotic deformity and new pain from the recompression of the cemented vertebrae. Consequently, it has significant importance of investigating the risk factors for such height loss result from the cemented vertebrae; nevertheless, few reports discussed the risk factors of significant vertebral height loss after PVP, and many results have been controversially caused by the limited data and a lack of the unique standard measurement of vertebral height and evaluation for the rate-of-change of vertebral height [7, 8].

It has a broad consensus that recompression of an augmented vertebra could frequently occur after PVP, and the probability of recompression is increasing with the increasing application of PVP [9, 10]. The representative risk factor associated with recompression after PVP is osteoporosis; other factors associated with the vertebral height loss include the cemented volume, advanced age, bone mineral density, the distribution pattern of cement injected into the fractured vertebra, and contact between bone cement and upper-lower endplates [9, 11, 12]. However, these studies did not include such vital factors associated with osteoporosis as bone turnover markers, including parathyroid hormone (PTH), C-terminal telopeptide of type I collagen (β-CTX), N-terminal propeptides of type I procollagen (PINP), osteocalcin (OC), serum 25-hydroxy vitamin D3 (25-OH-D3) levels, and alkaline phosphatase (ALP). Bone turnover markers are parameters reflecting the dynamic status of osteoporosis. This means that the bone mineralization and bone transformation in OVCF patients can be indirectly understood by detecting serum bone metabolism indexes. The authors hypothesized that there was a certain correlation between bone turnover markers and postoperative cemented vertebral height loss. Therefore, this study aimed to investigate the association of the risk factors, including bone metabolism markers, with the cemented vertebra recompression. It was also the first report in the literature to study the correlation between serum bone metabolism indexes and cemented vertebra recompression, providing clues for further clarifying the risk factors of cemented vertebra recompression.

## Materials and methods

### Study participants

Of those subjects who suffered from fresh compression fracture on only one spinal segment between May 1, 2016, and March 1, 2019, at the institution of Spine Center in our hospital, 109 consecutive patients were analyzed. Sixteen patients were excluded from the study, including three patients who died of unrelated disease within two months, five patients who underwent new vertebral fractures observed at adjacent levels, and eight patients who had not enough X-ray radiographs and refused to take examinations at our outpatient clinic. All patients had used thoracic-lumbosacral orthosis after PVP for more than one month and took medication prescribed for osteoporosis (Alendronate Sodium and Vitamin D3, one tablet/week) and kept proper comparable radiological images during the time of follow-up. This study was approved by the Institutional Ethics Committee of Huadong Hospital Affiliated to Fudan University. All participants in the study received written and oral information prior to giving written consent, and the study was performed following the Declaration of HELSINKI.

### Inclusion and exclusion criteria

Inclusion criteria were as follows: (1) single-level OVCF and the fractured segment was at T6 or lower; (2) fresh fractures that all patients’ collapsed vertebral bodies had the presence of bone marrow edema on magnetic resonance imaging (MRI) T2-weighted short tau inversion recovery sequences; (3) age over 60 years; (4) no severe surgical complications, for example, post-operative neurologic deficit because of the cement leakage; and (5) patients’ back pain caused by OVCF with ache on percussion of the fracture vertebral spinous position. Exclusion criteria were (1) non-osteoporotic vertebral compression fracture related to pathologic issue, infection, and malignancy; (2) multiple-level OVCFs; (3) severe significant neurologic deficit before and after surgery; (4) life-threatening complication after PVP, such as systemic infection, pulmonary embolism, or hypostatic pneumonia; and (5) occurrence of new vertebral fracture observed at adjacent levels after PVP.

### Surgical procedure

PVP was performed within three days of hospitalization for all patients. The previous report described the operation previously in detail; besides, every operation was conducted by the specified spinal surgeons with sufficient clinical experience, and the skills designed by Garfin et al. [13]. The patients first placed in a prone position on the operating table were administered under local anesthesia (2% lidocaine). With the guidance of two single-plane mobile C-arms, the anterior–posterior and lateral views of the fractured vertebra were confirmed. After incision of the skin, two 11-gauge needles were placed parallel to the superior and inferior edges of the pedicle, percutaneously into the anterior part of the vertebral body with a transpedicular or perpendicular approach. The injection of polymethylmethacrylate (PMMA) cement (Stryker, Kalamazoo, USA) into the fractured vertebral body was ceased until the cement reached the posterior one-fourth of the body or if PMMA extravasated outside the bone. The volume of bone cement inserted during the operation for each vertebra was recorded. Thoracic-lumbosacral orthosis were supplied to all patients for one month, and osteoporotic medications were used postoperatively.

### Radiological data collection and assessment

To analyze and evaluate the radiological results of PVP, pre-operative, immediately post-operative, and final follow-up X-ray radiographs of each patient were collected. The follow-up period was no more than one year. The anterior body height of the fractured vertebrae and the upper and lower adjacent fractured vertebrae were measured in lateral X-ray films. The prone lateral radiographs were substituted for standing lateral radiographs at three point-in-time of pre-operative, immediately post-operative, and the last follow-up because of film unavailability. Vertebral height measured immediately post-operative maybe not be under the same condition when measured on the last follow-up radiograph because of magnification error created by inconsistencies in patient positioning and tube-to-film distance. Because the original height of fractured vertebrae was closer to the average anterior height of the upper and lower adjacent vertebrae [14], in order to more accurately reflect the height changes of the vertebral body, we introduced an index of the anterior vertebral height ratio(AVHR) which was expressed as a percentage of the fractured vertebral height divided by adjacent fractured vertebral height instead of an absolute numerical value of the fractured vertebral height in our previous study [15]. The fractured vertebral height loss ratio (FVHLR) was calculated by subtracting the immediately post-operative AVHR from the last follow-up AVHR. All the height values were measured twice by two radiologists individually and independently in order to eliminate intra-and inter-observer bias to the utmost. All radiologic measurements were checked digitally using the picture archiving and communication system.

### Grouping and clinical study

The cemented vertebra recompression after PVP was regarded as the FVHLR ≥ 15% during follow-up [11, 16]. Patients who did not meet the above criteria within a one-year follow-up period were considered non-recompression after PVP. According to the above standard, the participants were divided into 32 patients in the recompression group and 61 patients in the non-recompression group. The scores of the total hip bone mineral density (BMD) of each patient were measured using dual-energy X-ray absorptiometry to determine the extent of osteoporosis before the operation, and the average BMD score was analyzed by excluding the BMD scores of the patient who differed by more than one standard deviation from the other one. Laboratory investigations including serum bone alkaline phosphatase (ALP), serum of calcium and phosphorus, and bone metabolism markers were performed on the morning following hospitalization for all subjects. We, respectively, regarded PTH, β-CTX, PINP, OC, 25-OH-D3, and ALP as markers of bone turnover. Age, gender, BMI, the volume of bone cement injected, leakage of bone cement, fractured vertebra segment, contact between the bone cement and the endplates and the medication history were also documented.

### Statistical analysis

All analyses were performed using IBM SPSS software for Windows version 23.0 (SPSS, Chicago, IL, USA). Firstly, continuous variables (BMD, Serum calcium and phosphorus, ALP, Volume of bone cement injected, PTH, β-CTX, PINP, OC, 25-OH-D3 levels) were checked to determine whether they were normally distributed. As these variables were not normally distributed, Mann–Whitney U test was used to examine the association between these variables in patients with and without cemented vertebra recompression. We converted the continuous variables such as age and BMI into categorical variables. Univariate Logistic regression analysis was used to determine the association between the cemented vertebra recompression and each categorical variable. Variables with a significant probability of *P* < 0.25 were then included in the multivariate logistic regression analysis [17]. Corresponding 95% confidence limits (CIs) were reckoned with confidence interval estimation, and *P* < 0.05 was statistically significant.

## Results

### Univariate analysis of variables related to the cemented vertebra recompression after PVP

**Fig. 1:**
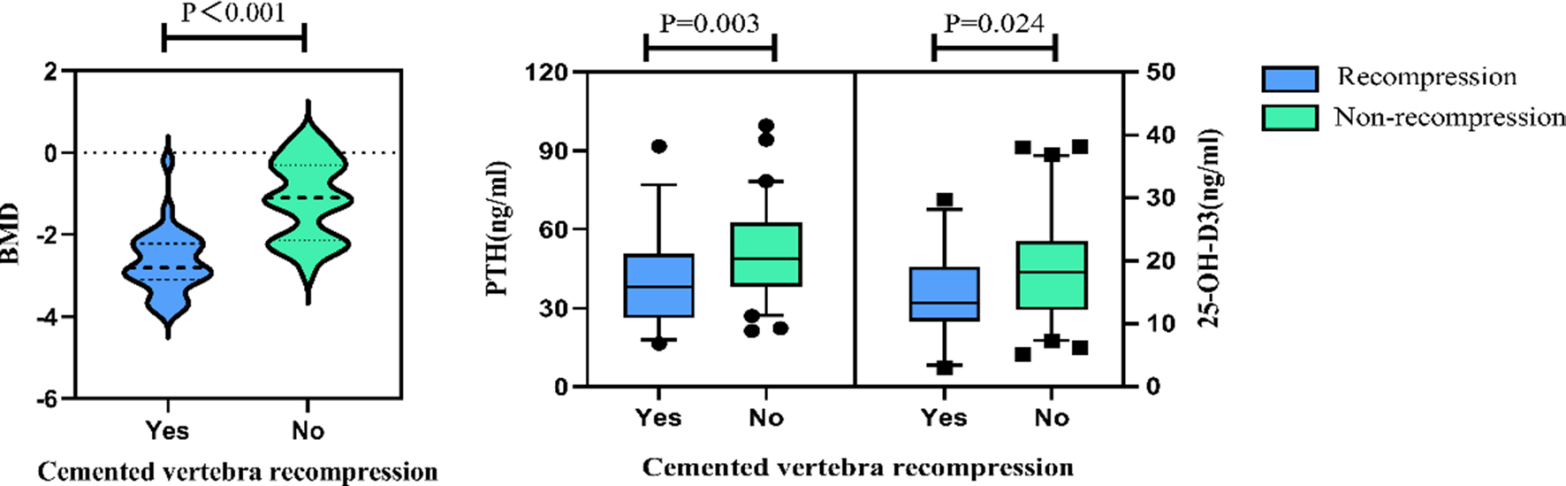
Comparison of BMD, serum PTH, and 25-OH-D3 between two groups (Group symbols: Recompression, blue color; Non-recompression, green color)

The continuous variance analysis was performed using Mann–Whitney U test, indicating that a significant difference has been observed between the recompression group and non-recompression group in BMD, serum PTH, and serum 25-OH-D3, and the difference was statistically significant (*P* < 0.05) (Fig. 1). The T value of BMD in the non-recompression group (median = -1.1, IQR = − 2.1 to − 0.3) was significantly higher than that in the recompression group (median = − 2.8, IQR = − 3.1 to − 2.25), and the difference was statistically significant (Z value = 6.189, *P* < 0.001). Similarly, the level of serum PTH and 25-OH-D3 in the non-recompression group were 48.9 pg/ml (IQR = 38.3–61.6 pg/ml) and 18.2 ng/ml (IQR = 12.3–22.9 ng/ml), respectively, and the level of serum PTH and 25-OH-D3 in the recompression group were 38.1 pg/ml (IQR = 26.7–50.3 pg/ml) and 13.3 ng/ml (IQR = 10.45–18.5 ng/ml), respectively, with statistically significant differences between the two groups (Z value = 2.952, 2.252, *P* = 0.003, 0.024). The specific analysis results are shown in Table 1.

**Table 1:** Comparison of continuous variables of patients between two groups (the Mann–Whitney U test)

Clinical parameters	Mean rank	Median	IQR	Z score	*P* value
*The volume of bone cement injected*					
Recompression group	45.55	5.5	4.5–6.0	− 0.381	0.703
Non-recompression group	47.76	5.25	5.0–6.0		
*BMD*					
Recompression group	23.11	− 2.8	− 3.1 to − 2.25	− 6.189	< 0.001*
Non-recompression group	59.53	− 1.1	− 2.1 to − 0.3		
*Serum calcium*					
Recompression group	43.08	2.22	2.2–2.3	− 1.070	0.285
Non-recompression group	49.06	2.2	2.1–2.3		
*Serum phosphorus*					
Recompression group	46.17	1.135	0.985–1.23	− 0.214	0.830
Non-recompression group	47.43	1.13	1.01–1.24		
*ALP*					
Recompression group	41.23	75	61.5–96	− 1.492	0.136
Non-recompression group	50.02	85	69–102		
*β-CTX*					
Recompression group	51.75	613.1	476–782.6	− 1.229	0.219
Non-recompression group	44.51	516.4	391–721.4		
*OC*					
Recompression group	48.91	15.25	9.9–21.3	− 0.493	0.622
Non-recompression group	46	13.4	9.7–18.2		
*P1NP*					
Recompression group	41.78	43.45	28.5–57.3	− 1.351	0.177
Non-recompression group	49.74	51.6	36.6–67.4		
*PTH*					
Recompression group	35.59	38.1	26.7–50.3	− 2.952	0.003*
Non-recompression group	52.98	48.9	38.3–61.6		
*25-OH-D3*					
Recompression group	38.3	13.3	10.45–18.5	− 2.252	0.024*
Non-recompression group	51.57	18.2	12.3–22.9		

* and bold indicate statistically significant findings

Univariate logistic regression analysis showed that the cemented vertebra recompression after PVP was correlated with whether the bone cement is in contact with the endplates, and the correlation was statistically significant (odds ratio 0.265, 95% CI 0.103–0.682, *P* = 0.006). There was no significant correlation in gender, BMI, age, fracture segment, bone cement volume, the medication history, and whether the bone cement is leakage, as showed in Table 2.

**Table 2:** Comparison of categorical variables of patients between two groups (Univariate Logistic regression analysis)

Clinical parameters	Cemented vertebra recompression				
	*B* value	SE value	Wald value	OR (95%CI)	*P* value
*Sex*					
female	0.279	0.584	0.229	1.322 (0.421–4.152)	0.632
male				1	
*BMI*					
overweight	0.280	0.447	0.393	1.321 (0.551–3.178)	0.531
normal weight				1	
*Age*					
super-advanced age(> 80 years)	0.433	0.440	0.967	1.542 (0.651–3.653)	0.325
advanced age(60–80 years)				1	
*Contact between bone cement and the endplates*					
YES	− 1.329	0.483	7.578	0.265 (0.103–0.682)	0.006*
NO				1	
*Bone cement leakage*					
YES	0.096	0.489	0.038	1.101 (0.422–2.871)	0.845
NO				1	
*Fractured vertebra segment*					
Thoracic region	− 0.431	0.554	0.604	0.650 (0.220–1.925)	0.437
Lumbar region	0.360	0.579	0.386	1.433 (0.461–4.456)	0.535
Thoracolumbar region				1	
*The use of antihypertensive drugs*					
Yes	0.172	0.439	0.154	1.188 (0.503–2.807)	0.695
No				1	
*The use of hypoglycemic drugs*					
Yes	− 0.255	0.546	0.219	0.775 (0.266–2.258)	0.640
No				1	
*The use of antiplatelet drugs*					
Yes	− 0.152	0.521	0.086	0.859 (0.309–2.383)	0.770
No				1	
*The use of antiarrhythmic drugs*					
Yes	− 0.817	0.823	0.986	0.442 (0.088–2.216)	0.321
No				1	

* and bold indicate statistically significant findings

### Multivariate logistic regression analysis of the factors related to the cemented vertebra recompression after PVP

Based on the result of univariate logistic regression analysis and Mann–Whitney U test, variables with a significant probability of *P* < 0.25 were put into the following multivariate logistic regression analysis. Therefore, seven variables, including BMD, ALP, P1NP, PTH, 25-OH-D3, β-CTX, contact between bone Cement and endplates, were included in the multivariate Logistic regression model. Among them, high BMD (odds ratio 0.089, 95% CI 0.026–0.306, *P* < 0.001) and high level of serum 25-OH-D3 (odds ratio 0.877, 95% CI 0.792–0.971, *P* = 0.012) were negatively correlated with the cemented vertebra recompression after PVP, which were the protective factors (*B* < 0). The specific analysis results are shown in Table 3.

**Table 3:** Risk factors related to the cemented vertebra recompression after PVP in OVCF patients (Multivariate logistic regression analysis)

Clinical parameters	*B* value	SE value	Wald value	*P* value	OR value	95%CI
Bone cement distribution	− 1.241	0.766	2.623	0.105	0.289	0.064–1.298
BMD	− 2.419	0.63	14.743	< 0.001*	0.089	0.026–0.306
PTH	− 0.027	0.024	1.274	0.259	0.973	0.929–1.02
25-OH-D3	− 0.131	0.052	6.332	0.012*	0.877	0.792–0.971
P1NP	− 0.005	0.017	0.077	0.782	0.995	0.963–1.029
ALP	− 0.027	0.014	3.679	0.055	0.973	0.947–1.001
β-CTX	− 0.001	0.002	0.41	0.522	0.999	0.995–1.002

* and bold indicate statistically significant findings

## Discussion

OVCFs are common among older Chinese adults, especially postmenopausal women due to bone loss caused by estrogen deficiency. The back pain, the kyphotic deformity, and the disability to be moved resulting from OVCFs have long motivated the search for effective techniques. As a result, PVP and percutaneous balloon kyphoplasty (PKP) have been suggested as effective and minimally invasive procedures for OVCFs [18, 19]. The PKP is an analogous technique to PVP, but it has an additional step of expanding balloons via bilateral transpedicular approach in the vertebrae to create cavities before injection of cement [20]. Some researchers have indicated that PKP is more effective and safer to restore the vertebral height than that of PVP [21]. However, we found that further recompression of cement vertebrae with significant vertebral height loss and aggravated local kyphotic deformity occurred more frequently in the patients after PKP during the follow-up period. Kim et al. [22] suggested that treated vertebral height loss was more prominent in PKP than in PVP, which was consistent with our clinical conclusion. They thought that PMMA filled the cancellous portion of the vertebrae with an interdigitated pattern in PVP, which was easier to transmit the load from the upper endplate to the lower one. In contrast, PMMA is not interdigitated but consists of one or two solid masses in PKP. Besides, the increased restoration of the fractured vertebral height by PKP could promote soft-tissue tension around the vertebrae, leading to an increasing loading adjacent to the cemented vertebrae, which can exacerbate the height loss of the treated vertebrae and the occurrence of adjacent vertebral fractures. Hence, we chose PVP to treat the OVCFs instead of PKP gradually.

It is widely accepted that the recompression of cemented vertebrae frequently occurred after PVP, typically within six months after invasive surgery based on our clinical observation. However, what are the critical factors related to inducing height loss of previously treated vertebrae after PVP is uncertain. Therefore, we conducted a detailed analysis of potential risk factors for height loss of cemented vertebrae, especially the bone metabolism markers, which are analyzed as the risk factors associated with cemented vertebral height loss for the first time.

The presence of non-PMMA-endplate-contact might play an essential role in inducing recompression after PKP treated vertebrae [9]. In addition, the distance between PMMA and endplate was an important risk factor for the recompression of cemented vertebrae after PKP, and the recompression of cemented vertebral height was positively correlated with the distance between PMMA and endplate [23]**.** Besides, Zhang et al. emphasized that the contact between bone cement and upper-lower endplates was an important independent protective factor for the recompression of cemented vertebrae after percutaneous vertebral augmentation [11]**.** Similar to the previous studies, this research indicated that the recompression of cemented vertebra after PVP was correlated with the contact between bone cement and upper/lower endplates (odds ratio 0.265, 95% CI 0.103–0.682, *P* = 0.006). Hence, we suggested that spinal surgeons might fill the treated vertebrae with adequate cement, which requires the cement should contact with upper-lower endplates during the surgical process. An et al. argued that the more bone cement injected, the lower incidence of augmented vertebra recompression occurred after PVP [9], while we found no correlation between bone cement volume and cemented vertebra recompression in this study.

High bone turnover was a risk factor for new fractures for patients with osteoporosis [24]. Nonetheless, there were few reports for analyzing the relationship between bone turnover and height loss of cemented vertebrae. Komemushi et al. [25] suggested that the combination of high levels of bone resorption markers and normal levels of bone formation markers might be associated with the increasing risk of new recurrent fractures after PVP. This research only concerned the association between bone turnover markers and new recurrent fractures but not the recompression of treated vertebrae; moreover, the number of their investigated subjects was only 30, and their bone turnover examination just included urinary β-CTX and serum ALP. In the present study, we considered the serum bone metabolism markers containing PTH, β-CTX, PINP, osteocalcin, 25-OH-D3 levels, and ALP as predictive risk factors for the cemented vertebral height loss. Our findings revealed that the level of serum PTH of cemented vertebra recompression group (median = 38.1 pg/ml, IQR = 26.7–50.3 pg/ml) was lower than that of non-recompression group (median = 48.9 pg/ml, IQR = 38.3–61.6 pg/ml, *P* = 0.003). As all we know, PTH could regulate the level of calcium in serum and increase bone formation through stimulating osteoblasts to produce local regulatory factors and regulating bone growth by mediating the proliferation and differentiation of osteoblasts [26, 27]. If applied intermittently, PTH could build new bone by constantly stimulating the receptor activator of the nuclear factor [28]. Yang et al. [29] found that PTH (1–34) was a safe and effective to improve vertebral BMD at onset time, growth rate and range in the treatment of primary osteoporosis, which could increase the bone mass of patients and reduce the risk of fracture. Likewise, Ban et al. [30] divided 90 elderly male hip fracture patients into the effective and the delayed healing groups according to the final healing results and found that the serum thyroid hormone level of effective healing group was significantly higher than that of delayed healing group. On the other hand, however, PTH could stimulate osteoclast activation to promote the release of bone calcium and phosphorus into the blood, ultimately increasing the bone resorption process and reducing bone mass [31]. In addition, hyperparathyroidism, caused by excessive secretion of PTH from the parathyroid glands and characterized by elevated serum levels of calcium and PTH, often results in increased bone resorption and a higher risk of osteoporosis and fracture [32]. Some researchers have reported that serum PTH concentration is negatively correlated with BMD, suggesting that higher levels of serum PTH mainly leads to bone catabolism and decreases BMD [33, 34]. Notably, although we found a significant difference in the level of serum PTH between the cemented vertebra recompression group and non-recompression group, there was no correlation between serum PTH level and cemented vertebra recompression after PVP in the multivariate logistic regression analysis, suggesting that serum PTH might be a confounding factor. Therefore, the relationship between PTH levels and bone resorption/formation remains unclear.

Vitamin D plays a vital role in regulating blood calcium levels and maintaining blood phosphorus levels. Calcium and phosphorus are involved in vitamin D metabolism and regulate blood calcium levels by affecting intestines and kidneys [35]. In addition, vitamin D deficiency is associated with fragility fractures, osteoporosis, osteoporotic fractures, muscle weakness, and body balance [26]. This study found that serum 25(OH)D level of cemented vertebra recompression group (median = 13.3 ng/ml, IQR = 10.45–18.5 ng/ml) was significantly lower than that of non-recompression group (median = 18.2 ng/ml, IQR = 12.3–22.9 ng/ml, *P* = 0.024). Moreover, a high level of serum 25-OH-D3 (*P* = 0.012, OR = 0.877) was negatively correlated with the recompression of cemented vertebra after PVP. Angeles et al. also found that low 25(OH)D serum levels were related to developing new vertebral fractures, suggesting it was necessary to correct vitamin D deficiency prior to performing vertebroplasty [36]. Vitamin D plays an important role in treating OVCF; thus, patients with OVCF at a lower level of serum vitamin D might have a greater risk of cemented vertebral height loss. In addition, we provided more physical defense measures for patients with low levels of vitamin D to prevent the height loss of cemented vertebral as much as possible, such as extending the fixation time of thoracic-lumbosacral orthosis and reducing daily weight-bearing activities. Therefore, we suggested that vitamin D supplementation was necessary for fracture healing and cemented vertebra height maintenance.

As a consensus, decreased BMD is a general known risk factor for fractures, and recent studies have found that decreased bone density is the leading cause of re-fracture of cemented vertebra and adjacent vertebra after PVP [37–39]**.** Similar to the previous researches, we found that the T value of BMD of cemented vertebra recompression group (median = − 2.8, IQR = -3.1 to − 2.25) was lower than that of the non-recompression group (median = − 1.1, IQR = − 2.1 to − 0.3, *P* < 0.001). In addition, high BMD (*P* < 0.001, OR = 0.089) was negatively correlated with the recompression of cemented vertebra after PVP, meaning that patients with decreased BMD had a high risk of cemented vertebrae recompression. Besides, Yang et al. [40] also reported that decreased BMD was a decisive risk factor for persistent back pain after PVP in OVCF patients. Therefore, BMD might play an essential role in the height loss of treated vertebrae, and positively anti-osteoporosis treatment is needed for elderly OVCF patients after PVP.

## Limitations

The present study has its limitations due to the number of subjects is not large based on the strict exclusion and inclusion criteria for patient selection. Moreover, the follow-up period is relatively short, while a longer follow-up time is necessary to determine the risk factors for height loss of treated vertebral bodies after PVP. Some new technologies, such as combined intravoxel incoherent motion diffusion-weighted MR imaging and magnetic resonance spectroscopy, have not been employed to differentiate osteoporotic fractures from osteolytic metastatic vertebral compression fractures in this study [41]. Finally, every patient’s daily activity is not controlled entirely in follow-up period after PVP. As the intensity of their daily activities increases, the mechanical pressure significantly increased upon cemented vertebrae, which might accelerate fractured vertebral height loss. Well-designed research using extensive materials would be helpful in terms of further evaluating the height loss of cemented vertebrae after PVP.

## Conclusion

In light of our results, we concluded that decreased BMD and lower level of serum 25-OH-D3 might be two critical risk factors for the height loss of cemented vertebrae after PVP. For elderly patients with OVCF undergoing PVP surgery, it is necessary to detect the level of serum 25-OH-D3 before surgery, which avails for evaluating and predicting the risks of postoperative cemented vertebra recompression.

## Data Availability

The datasets used and analyzed during the current study are available from the corresponding author on reasonable request.
